# Whole-genome sequence of *Geobacillus thermodenitrificans* K1041, a genetically tractable strain representative of the genus *Geobacillus*

**DOI:** 10.1128/mra.00848-23

**Published:** 2023-12-08

**Authors:** Saori Kani, Hirokazu Suzuki

**Affiliations:** 1 Faculty of Engineering, Tottori University, Tottori, Japan; 2 Center for Research on Green Sustainable Chemistry, Tottori University, Tottori, Japan; University of Strathclyde, Glasgow, United Kingdom

**Keywords:** *Bacillus*, thermophile

## Abstract

We present the whole-genome sequence of *Geobacillus thermodenitrificans* K1041, a bacterium that was originally identified as a genetically tractable thermophile. The genome consists of a circular chromosome that contains 3,848 genes. The sequence has a total size of 3,755,826 bp with a GC content of 49.18%.

## ANNOUNCEMENT

*Geobacillus* spp. comprise Gram-positive, rod-shaped, and moderately thermophilic bacteria. *Geobacillus thermodenitrificans* K1041 was originally identified from laboratory stocks as a strain that can efficiently accept exogenous plasmids via electroporation (1). We previously analyzed draft genome sequences of this organism (2). Herein, we present the whole-genome sequence of *G. thermodenitrificans* K1041.

*Geobacillus thermodenitrificans* K1041 was obtained as a lyophilized sample from the RIKEN BioResource Center (Tsukuba, Japan; no. RDB00139). The cells were cultured at 60°C using lysogeny broth under aerobic conditions. Genomic DNA was extracted from the cells using a cetyltrimethylammonium bromide-based method (3) and solubilized in 1 mL of a buffer that contained Tris-HCl at pH 8.0 (10 mM) and EDTA (1 mM). Genomic DNA was then purified from an aliquot of the resulting solution using NucleoSpin gDNA Clean-up kit (Takara Bio, Otsu, Japan).

The genomic DNA was sequenced using the PacBio and Illumina PE150 platforms. Default parameters were used for all software. The genomic sample was sheared into 10–15 kb using a g-TUBE device (Covaris, Massachusetts, MA, USA) and used to construct a long-read library using SMRTbell Express Template Prep Kit version 2.0 (Pacific Biosciences of California, Menlo Park, CA, USA). The library was sequenced using PacBio Sequel IIe system (Pacific Biosciences of California). The data were analyzed using SMRT Link version 11.1 (Pacific Biosciences of California) to yield 29,581 Hifi reads (average length, 7,982 bp; N_50_ length, 10,116 bp). The reads were assembled using Hifiasm version 0.13-r308 (4) and Canu version 1.7 (5) to give an assembly sequence. The sequence was circularized and rotated to start from the *dnaA* gene using Circulator version 1.5.5 (6). To correct sequence errors in the assembly, the same sample of genomic DNA was sheared into <0.5 kb by sonication and used to construct a short-read library using NEBNext Ultra DNA Library Prep Kit for Illumina (New England BioLabs, Ipswich, MA, USA). The library was sequenced as 150-bp paired-end reads on NovaSeq 6000 system (Illumina, San Diego, CA, USA). The analysis yielded 18,386,082 raw reads, which were validated using Fastp version 0.23.0 (7) to obtain 18,259,058 clean reads. In reference to these reads, the assembly sequence was polished using Pilon version 1.22 (8).

The resultant assembly consisted of one contig that contained 3,755,826 bp with a GC content of 49.18%. The genes were predicted using the National Center for Biotechnology Information Prokaryotic Genome Annotation Pipeline version 6.6 (9). The analysis suggested the presence of 3,848 genes, including 3,608 protein genes, 117 pseudogenes, 88 tRNAs, 30 rRNAs, and 5 ncRNAs, in the chromosome. In addition to *G. thermodenitrificans* K1041, *G. thermodenitrificans* T12 can be efficiently transformed via electroporation (10). As efficient electroporation is uncommon in *G. thermodenitrificans* (11), a comparative genome analysis of the K1041 and T12 strains with electroporation-inefficient strains may elicit a gene responsible for efficient electroporation of *Geobacillus* spp.

## Data Availability

The whole-genome sequence has been deposited at DDBJ/ENA/GenBank under the accession number CP133461. The raw reads have been deposited in the Sequence Read Archive under the accession numbers SRX21482915 and SRX21482916.
